# The genesis of the teacher habitus—a longitudinal study with Swiss primary teacher students

**DOI:** 10.1007/s35834-022-00350-w

**Published:** 2022-08-23

**Authors:** Julia Košinár, Anna Laros

**Affiliations:** 1 University of Applied Sciences and Arts Zurich, Zurich, Switzerland; 2 Zurich University of Teacher Education, Lagerstrasse 2, 8090 Zurich, Switzerland; 3 Institute of Primary Education, University of Applied Sciences and Arts, Northwestern Switzerland, Bahnhofstrasse 6, 5210 Windisch, Switzerland

**Keywords:** Studierenden- und Lehrpersonenhabitus, Längsschnittstudie, Dokumentarische Methode, Berufsbiografische Professionalisierungstheorie, Student and teacher habitus, Longitudinal study, Documentary method, Biographical professionalization theory

## Abstract

The teacher habitus concept offers a theoretical figure for the classification and interpretation of professionalization processes—especially from a vocational-biographical perspective. Milieu of origin and school biography are important for the genesis of the teacher’s habitus, since images of school life are already created in them, which are relevant for later professional action in the form of implicit orientations and explicit ideas. In the article, interview data from a longitudinal survey (3t) of primary teacher-students (*N* = 24), which were analyzed using the documentary method, are related to this concept. It turns out that the school biography is used as an argument for the choice of study, and frames the experience in the first internship. In the further course of the study, the professional requirements are increasingly classified against the background of one’s own skills and their concepts of the teaching profession. It can be seen, that students deal with requirements and arising crises and address their practice teacher in a very diverse way. Types of students and their habitus were identified at each time of the survey. For t2 (end of 2nd year) these were distinguished as: delimitation, extension, development, exploration and probation. On the basis of two contrastive case analyzes of the types “extension” and “development” it is shown how important one’s own school experience is and how differently the genesis of a teacher’s habitus takes place. The results illustrate the relevance of a (school) biographical reflection that should be given space to enable future teachers to access their implicit, action-guiding orientations and to make their own student habitus accessible to reflection.

## Abstract (de)

Das Lehrerhabituskonzept bietet für die Einordnung und Interpretation lehrberuflicher Professionalisierungsprozesse eine gehaltvolle Theoriefigur. Dem Herkunftsmilieu und der Schulbiografie werden für die Genese des Lehrerhabitus Bedeutung zugewiesen, da in ihnen bereits Bilder des Schulischen angelegt werden, die für das spätere berufliche Handeln in Form impliziter Orientierungen sowie expliziter Vorstellungen relevant sind. Im Beitrag werden Interviewdaten aus einer Längsschnittbefragung (3t) von Primarschulstudierenden (*N* = 24), die mit der Dokumentarischen Methode analysiert wurden, an diese Konzeption angelegt. Es zeigt sich, dass die Schulbiografie insbesondere beim Studieneintritt als Argumentationsfolie für die Studienwahl herangezogen wird und das Erleben im 1. Praktikum rahmt. Im weiteren Studienverlauf werden zunehmend die beruflichen Anforderungen vor dem Hintergrund eigener Fähigkeiten und Berufsbilder eingeordnet. Anforderungen und sich konstituierende Krisen sowie die Adressierung der Praxislehrperson ist sehr divers. Je Erhebungszeitpunkt wurden Studierendentypen und ausbildungsbezogene Habitus identifiziert. Für t2 wurden diese unterschieden als: 1. Abgrenzung, 2. Erweiterung, 3. Entwicklung, 4. Erkundung und 5. Bewährung. Anhand zweier kontrastiver Fallanalysen der Typen „Erweiterung“ und „Entwicklung“ wird aufgezeigt, welche Bedeutung der eigenen Schulerfahrung zukommt und wie unterschiedlich sich die Genese eines Lehrerhabitus vollzieht. Die Ergebnisse verdeutlichen die Relevanz einer (schul-)biografischen Reflexion, die Raum geben sollte, um zukünftigen Lehrpersonen Zugang zu ihren impliziten, handlungsleitenden Orientierungen zu ermöglichen und den eigenen Studierendenhabitus einer Reflexion zugänglich zu machen.

## Introduction

For some years now, the discourse on professionalization theory has been revitalized by explanations and reflections on the teacher habitus (Helsper 2018a, b, 2019). This concept includes, following the structural-theoretical paradigm, definitions of teachers’ professionalism and illustrates them as an “occupational” or a “professional habitus” (Helsper 2018a, p. 128ff.). In a model of the genesis of a teacher habitus, Helsper (2019) has mapped the importance of biography and milieu for the process of forming teacher habitus that can be used for theoretical differentiations (Kramer and Pallesen 2019) as well as a framework for case reconstructions. The vocational-biographical approach also finds in the concept of teacher habitus a justifying framework for various empirical phenomena and phenomena recognizable in educational practice (Košinár 2021). For example, taking into consideration the milieu of origin and school biography, provides a rationale for the stability of orientations: e.g. experiences of learning—success or failure—from the school context shape the image of schooling and of teachers far beyond the school years. Thus, it can be assumed that these experiences are carried over into teacher education, where they are reactivated as implicit knowledge in school internships, and become a guideline for action.

Due to the still-narrow empirical basis, this is only an assumption; but a large number of studies on teachers’ beliefs confirm the influence they have on decisions, students’ perceptions and their actions (e.g., Oser and Blömeke 2012; Wilde and Kunter 2016). Consistently, early acquired beliefs are associated with low potential for change. Low transformation potential is also confirmed by studies on teaching orientations and habitus (Hericks et al. 2018, Wittek et al. 2020, Košinár and Laros 2020). Until now, only few longitudinal case studies were available that could provide insight into process trajectories (for an overview: Košinár and Laros 2021).

Our paper addresses the aforementioned desiderata and uses longitudinal analyses of student cases to shed light on the genesis of teacher habitus during the period of study and with a focus on practical trainings. Thus, the span between a pupil habitus and a teacher habitus is considered, which still raises not only empirical but also theoretical questions. To clarify these, conceptual outlines of teacher habitus and its genesis are presented (2), followed by an outline of the state of research on student habitus (3). After introducing our project (4), we first provide a summary overview of key findings from our longitudinal study (5), before outlining developmental processes based on two contrastive cases that provide us with information about potential habitual transformations and localizations among a pupil habitus, student habitus or teacher habitus (6). The paper concludes with a summary of the findings and a discussion (7).

## Theoretical assumptions about the genesis of a teacher habitus

The concept of teacher habitus has its origins in Bourdieu’s concept of cultural theory (e.g., 1993). Helsper (2018a, p. 120) differentiates four “habitus configurations”^1^: the “familial primary habitus,” the “acquired individual habitus” and the teacher and pupil habitus, which he characterizes as “partial habitus forms” due to their field-specificity. The relationship of these four habitus, which become relevant in different biographical periods, “is to be conceived as a process” (ibid., p. 127). In the oeuvre of Bourdieu, the “question of the life-historical development and transformation of the habitus remains rather unresolved” (ibid., p. 120). This highlights the need for, and at the same time the innovative content of, an empirically supported approach to these processes, which have so far essentially existed as theoretical concepts.

In this, the pupil habitus has a significance that has not yet been precisely clarified. The “pupil habitus is rooted […] in the educational and school orientations of the family environment. However, the pupil habitus only finds its concrete form in the confrontation with school requirements in the context of concrete school cultures in the course of the school years” (ibid., p. 127). In this process, pedagogical and subject-specific orientations become embedded. They form negative and positive counter-horizons of school, teaching and teachers, which hint at the later teacher habitus as a “first silhouette” (ibid., p. 125) or as “raw forms and images of the teacher” (Kramer and Pallesen 2019, p. 81).

As Helsper (2019) points out, a transformation from pupil to teacher habitus should not be understood as a linear process. Kramer and Pallesen (2019, p. 80) justify this factually with the respective logic of requirements, which “cannot be thought of as identical” because the school and classroom requirements clearly differ from the pupil and teacher perspective. Thus, they characterize “the bringing forth of a teacher habitus […] as an independent and transformational process” (ibid.), in the execution of which that “ability to act [is] gained and maintained which is related to the structural demands of the teaching profession” (ibid., p. 81).

According to Helsper, the genesis of the teacher habitus takes place over the professional biographical phases of study, traineeship and career entry. During studies, “first new experiences with the school field emerge in the form of practical trainings” (Helsper 2018a, p. 126). Here, a first change of perspective from the pupil habitus to the perception of and dealing with the specific demand logic of a teacher takes place (cf. Chap. 5). During the teacher traineeship^2^ and the entry into the profession, one’s own orientations and practices are formed in “a specific school form, school culture, and its collegium” (ibid.). The formation of the teacher habitus in the sense of an “experience-saturated own professional understanding and the emergence of school and instructional action routines” (ibid.) needs at least several years.

As the explanations make clear, the initiation of the teacher habitus during studies remains vague. While Helsper (2019), Kramer and Pallesen (2019) follow a field-specific developmental logic in their explanations, empirical studies indicate that steps in the formation of a teacher habitus can already be identified during studies, although there is a great diversity. This diversity can be explained only in part by individual lines of development (e.g., age, previous professional experience, developmental orientation) or country-specific training conditions. It is strongly nurtured by (increasing) employment in schools during studies. Another argument for the fact that a teacher habitus is already initiated during study lies in the dissociation of study and practice components. Current findings (cf. 2.) on the student habitus can fuel the controversial discussion about the interlocking of theory and practice elements during study with concrete problem indications.

## State of research on student habitus

The number of reconstructive studies investigating the study period (e.g., Košinár and Laros 2018; Liegmann and Racherbäumer 2019) is limited. Currently, a whole series of projects and qualification works are in progress, which deal—predominantly by means of the documentary method—with the reconstruction of student habitus. In the following, two projects will be mentioned whose design and findings are related to the project on which this article is based.

With a focus on the study-entry-phase, the Hildesheim project “Individual Developmental Trajectories of Professionalization in the Teaching Profession—A Reconstructive Longitudinal Study on the Formation of the Teacher Habitus” (IndEL) will reconstruct the impact of the pupil habitus (or a professionally socialized habitus) on the genesis of a student habitus and the later teacher habitus. For this purpose, interviews with students with and without previous occupation (*N* = 8) are conducted at two points in time. The survey takes place before the first and after the second semester of study, between which the first internship takes place. As initial findings show, there is a tendency for students to “mentally” skip the study phase “as a professional development phase” due to the early practice of teaching (Kowalski 2022). The author points out that the missing formation of a scientific-reflexive student habitus, limits students’ opportunities for a reflexive distancing from their own pupil habitus.

This view is also shared by Kahlau (i.p.) in her dissertation, which researches the student habitus in the master’s program at the University of Bremen (*N* = 9). In her project “(De‑)Professionalization through School Practice Experiences” she elaborates on how students interpret and process demands in the *Praxissemester* and how they develop their understanding of their roles in the “in-between”. In the reconstructed “anticipated professional understanding” (Kahlau i.p.) of the students, divergent “characteristics of a student habitus” become apparent. There are differences between a “subject orientation” and a “social orientation”. For the former, “the subject is the core of professionalization”, while for the latter, the school “as a social situation is the decisive factor for the choice of profession and the understanding of professionalization”. With regards to the processing of developmental tasks, Kahlau identifies two types: “orientation to the new” and “orientation to the known.” Relating the typologies, the author reconstructs three student habitus, which she differentiates into “engaging in transition,” “persisting in pupil habitus”, and “anticipated teacher habitus”. She attributes the readiness to “relate reflexively to one’s own educational biography” most likely to the student habitus of “engaging in transition”.

The variety of studies currently in progress will enable teacher habitus research to present more differentiated (professional) biographical processes and transitions from the pupil habitus to the student habitus and the initiation of a teacher habitus and to specify these processes and transitions according to subject and topic.

## Presentation of the project: research design, methodology and key findings

The project “Processes of Professionalization of Primary Teacher Students—A Reconstructive Longitudinal and Multi-Level Analysis” (2017–2020, founded by SNF) investigates the genesis of a student and teacher habitus over time during the entire study program. In the Swiss teacher education, professional practice training within the (three-year) degree program is of central importance. Against the background of a generalist education of primary teachers (6–8 subjects), the internships are supposed to enable students to take up the task of a classroom teacher with a wide range of subjects later on.

The starting point for the present project is the reaccreditation of the study program and the new introduction of a one-year internship in partner schools. The practical phases at (institution anonym), consist of a three-week, closely supervised basic internship (first year), the partner school year (second year) and a four-week focus internship (third year) with a high degree of autonomy. The partner school year includes weekly internship-days and five weeks over two semesters. Partner school groups include six practicum teachers, 12 students and two lecturers (one subject didactician). Accompanying courses take place at the partner schools.

Our project connects to the vocational-biographical theory of professionalization. In addition to the habitus concept, the project is theoretically framed by the developmental task concept (Hericks 2006; Keller-Schneider and Hericks 2017; Leineweber et al. 2021), that is used as a heuristic for the qualitative classification of the recognizable processes over the survey phases. Furthermore the theory of experiential learning forms the background for the recording of professionalization steps. Here, a crisis or irritation is seen as the starting point for possible development. If previous certainties and routines of action are irritated, a learning energy can be triggered in the “tension between not-knowing and knowing, non-ability and ability” (Combe and Gebhard 2007, p. 48), which, as previous studies of ours show, is harnessed in very different ways (Laros and Košinár 2019).

**Fig. 1:**
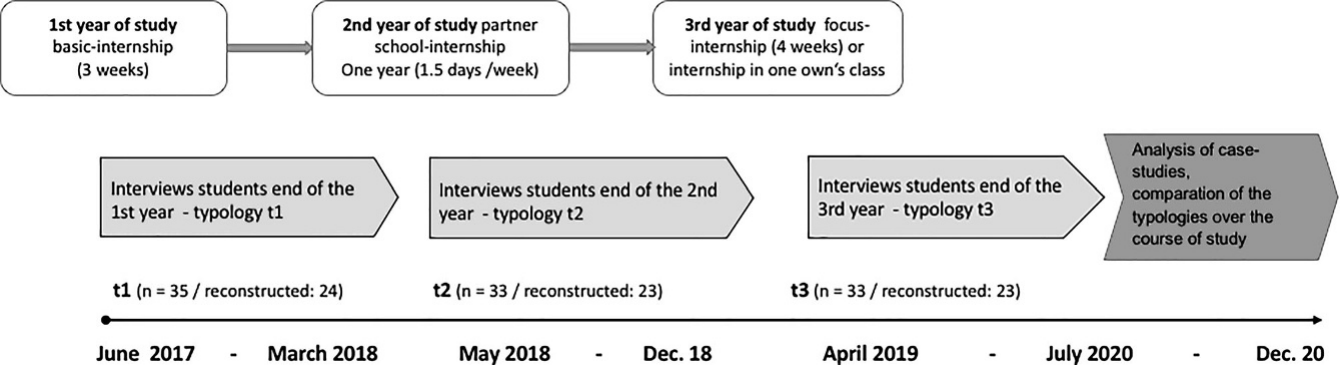
Research design of the interview sub-study

The project is composed of two sub-studies. The focus of this article is on the reconstructive longitudinal interview study. The sample consists of students (*N* = 35) who enrolled at the end of their first year of study at partner schools that participated as research schools. In three waves of data collection, they were interviewed at the end of each academic year (Fig. 1).

Our research interest comprised different topics and questions. The interviews focus on the students’ experience in their internships (especially in the classroom with the students), their collaboration with other actors (especially the practice teacher), and their handling of crises constituted by the requirements. In this article, we focus on their professional and professionalization-related orientations and the process as it is depicted over the three survey phases:

- What orientations emerge with regards to teachers’ professionalism and professionalization? To what extent do these change in the course of the studies?
- Which transformation processes of student orientations and their habitus can be identified over the longitudinal section?

In the interviews, narration-generating impulses were set at the beginning, which were oriented toward the respective professional practice phase. While at t1 the path to study was only briefly touched, at t3 a more detailed school biographical narrative was initiated.

### Data analysis with the documentary method

The guiding principle for data analysis was the documentary method (Bohnsack 2017). This method distinguishes between communicatively generalized, explicit knowledge and conjunctive, implicit knowledge, which is mostly not reflexively accessible to the actors and is reflected in their practices of action as well as in their narratives. The methodological procedure aims at reconstructing the conjunctive practices. During analysis, attention is first paid to how the topic area on which the interviewees express themselves is structured by themselves. This is elaborated in the context of the *formulating interpretation* and aims at WHAT the respondents say about the topic. In a second evaluation step, the *reflective interpretation*, we look at HOW the individual previously identified topics are framed and negotiated. In a further step, the reconstructed orientation frames of the cases are related to each other and a typology is being reconstructed. In our project, 24 interviews (t2, t3: 23) were selected from the total sample and initially analyzed as individual cases per survey wave.

The analyses were based on comparative dimensions (Table. 1) developed from the research interest and the data material, which formed the structure for the case analyses. The reflective interpretation of each dimension led in each case to a conclusion, in which an attempt was made to capture the orientation framework. The comparison between the cases was made via the conclusions, which were bundled into types.

**Table 1:** Comparative dimensions of the case analyses and comparison—modified phase-specifically

	T1 (entry into studies)	T2 (long-term internship)	T3 (focus internship)
*Comparative dimensions*	1. Orientation at study-entry	1. Orientation during long-term internship	1. Orientation during focus internship
	2. Meaning of the internship	2. Meaning of the internship	2. Meaning of the internship
	3. Interpretation of and dealing with crises	3. Interpretation of and dealing with crises	3. Interpretation of and dealing with crises
	4. Addressing of practice teacher	4. Addressing of practice teacher	4. Addressing of practice teacher
	5. Role of tandem partner	5. Role of tandem partner	5. Role of tandem partner

The typing was done as a cross-sectional typology (Košinár & Laros, 2021), i.e., it was conducted for each wave of the survey. Through this procedure, it became apparent that in each study year, a respective phase-specific orientation problem structured and framed the experiences and actions of the interviewees:

- 1st year of study: Entry into studies as a transition with connection to one’s own school and professional biography.
- 2nd year of study: professional socialization process (in the long-term internship) between student and intern habitus
- 3rd year of study: Continued process of socialization between interns and teachers with the aim of proving oneself in the upcoming career entry.

The reconstruction of the orientation framework (modus operandi) in dealing with the phase-specific problem was summarized as a basic typology or, in terms of professionalization theory, as a “development typic” (Bohnsack 2017). We approached the habitus of the students via reconstructing their dealing with the phase-specific problem (ibid.). The comparison of case studies and typologies in longitudinal section made it possible to work out stabilities, modifications, and transformations.

### Summary of central results of the typologies in the longitudinal section

In the following, we present key findings from the three cross-sectional typologies. Subsequently, two case analyses in the longitudinal course are presented as examples in chapter five.

With regards to the concept of the genesis of the teacher habitus, our findings confirm that (school) biographical experiences unfold interpretive power in the early phase of studies. This can be found, for example, in students’ narratives on how they chose their study or profession. Two orientation frames can be distinguished at the time of study entry: “profession exploration” and “profession decision”. Against the background of the respective (school) biographical experiences, “profession exploration” takes place either under high pressure to fit (Type Ia), in the mode of openness (Type Ib), or in the sense of a determination of one’s position in an occupation that has been envisaged for a long time (Type II). Students who frame their studies as a “profession decision” have already completed a negotiation process—sometimes lasting years—in advance.

For those who have a professional career behind them, the study decision takes place in a positive counter-horizon to the former job (Type IIIc), for those who have negative school experiences, in a positive counter-horizon to their own experiences (Type IIIa). A third group has first critically examined the biographical self-evidence, which is also found in Type II (Type IIIb).

The respective orientation at the beginning of the study also frames the first internship, which is interpreted and processed as a learning or probationary situation against the background of previous school and learning experiences. The respective orientation has a recognizable effect on the addressing of the practice teacher, the tandem partner and on the handling of (crisis-like) demands (Košinár 2022).

However, the influence of prior (school) biographical experiences seems to weaken in the course of the study. As the interview analyses show, with the long-term internship (in the second year of study), the students’ focus increasingly shifts to the requirements that become visible in everyday school life, with which they enter into a process of negotiation. In the attempt to react to the professional demands in a way that corresponds to the expectations of the instructors and/or the necessities of the teaching process, options for action are applied, rejected or confirmed. This takes place differently, depending on the orientation of the internship. We were able to identify five types, which, very briefly, can be distinguished as follows:

1. delimitation type, which seeks ways to reduce the demands placed on it as much as possible (strategically or out of overwhelm)
2. extension type, who actively uses the possibilities of the internship, following above all his own ideas and preferences.
3. development type, who works specifically on professional requirements and own development goals, even if this means to work on crises of own action limitation.
4. exploration type, who explores the future professional field with positive interest, without taking full responsibility for their own actions or crises.
5. probation type, who is closely oriented to the practice teacher and fulfills her tasks, tries out her professional actions.

This distinction of types is found again at the end of the study (t3). Here, too, five types were identified and most cases end up in the structurally identical type, which empirically confirms the stability of the training- and school-related habitus. With the imminent career entry, the focus in some types changes more strongly in the direction of coping with professional requirements, which is expressed as personal responsibility (5.1 Lasnic case) or as a final skills test (5.2 Summer case). In other types, especially in the probation type, the orientation toward the requirements and feedback of the practice teacher remains stable. This recognizable range among the graduates can be partly explained by the fact that some of them are already working as substitutes in schools. However, as the following case studies show, their respective orientations and “habitus formations” (Kramer and Pallesen 2018, p. 42) shape their job-related actions differently.

## Case studies

In the following, highly abbreviated case analyses of representatives of the extension (Noah Summer) and the development (Tina Lasnic) types are presented. Both types enter into an active process of negotiation with professional demands and have already worked as substitutes at schools during their studies. However, clear differences can be found with regards to a reflexive processing of crisis-related experiences and thus also to their student habitus. Divergences can also be identified with regards to the process of forming a teacher habitus, whereby it can be shown that both students connect to their school experiences.

### Case Tina Lasnic

Tina Lasnic (TL) is 20 years old at the beginning of the study. After graduating from high school in Germany, she comes to Switzerland for a one-year internship at a Montessori school. This intermediate year is intended to serve as a “break” before a planned study of languages and economics.

At the end of the internship, TL revises her original choice of studies and starts studying to become a primary teacher at (institution anonymous). She completes her three-week block internship in the 1s t year of study at an bilingual elementary school. Her partner-school internship in the 2nd year takes place in an inclusion class in a multi-professional team, where she also completes her focus internship in the third year in her own class as a substitute. At the time of the last interview, she has a substitute position at another school in addition to her studies and will have her own classroom after the summer vacation.

TL’s time in high school carries negative connotations. She obviously associates authority and power with the “teacher position” and initially distances herself from this career choice.

Tina L: I […] but never saw myself in this teacher position just as a policeman and correcting homework ,has everyone his homework’, and so on. (t1_TL, 18–19)

In the interview at t3, we learn that TL is the first in her family and her circle of friends to attend a *Gymnasium*. This milieu ascent causes an experience of strangeness that shapes the entirety of her school years. Thus, the scary behavior of a single teacher (as it turns out at t3) is enough to be generalized in retrospection and almost referred to the entire teaching staff.

Tina L.: I actually only had a great teacher in elementary school and then in between certainly again but mostly quite authoritarian black-white, wrong-right teachers who somehow made me very afraid to think freely or to be able to express myself freely, so I always had the feeling they expect a certain answer from me, when they ask me now and if that does not correspond to their expectations it is automatically wrong, and I had the feeling I had little free space really or little chances to be accepted and my thinking processes to be appreciated. (t1_TL, 50–59)

In her school-related narratives, the silhouette of a teacher habitus of suppression and non-recognition is indicated. It is documented across the three interviews as a positive counter-horizon of the *recognition pupils in their individuality and their appreciation or the avoidance of shaming*. This central pedagogical orientation becomes visible as a homology in various sequences in the course of the survey, in which TL’s interpretations of her teaching and her actions in practice can be traced. Initially from the observer perspective in the internship at the Montessori school, she recognizes that such positive teacher action is possible in the regular school system. Here, her perception and interpretation schemata, which are incorporated in the student habitus, are irritated: She experiences* new spaces of possibility in the teaching profession*, which show compatibility with her frame of orientation. In this way, a *transformation of her professional image* takes place on an explicit level. This happens between the end of her school years and the end of her first year of study.

Tina L.: and through the internship my image of the teacher changed a lot and then of course during my studies, and also now just didactically somehow, I actually noticed that I had quite a few teachers who simply portrayed that and not necessarily that that is the only right thing they did but that you have so many other possibilities to teach children something, and, yes, simply to be a teacher. (t1_TL, 43–47)

It is those new experiences in the school system that not only allow TL, but even make it constitutive, to examine the teaching profession for herself. Her internships are shaped by testing the viability of her ideas and ideals. At the beginning of the study, the practicum teacher is closely observed and used as a model in her own classroom management and in her interactions with pupils. Later, in the partner school year, TL tries to implement her pedagogical and didactic claims and experiences several times that she reaches the limits of her ability to act. In the process, a *reflexive development-oriented student habitus* reveals its contours—the crises are interpreted as an occasion for learning and, with the support of the practice teacher, TL works through them until they are resolved.

In the course of the long-term internship, other dimensions of her teaching habitus sharpen, in addition to her recognition orientation. In a multi-professional team in an inclusion class, TL encounters the requirement of cooperation at an early stage. As a planner and designer of learning environments, she assumes full responsibility for the learning success of all students and desperately wants to meet the children’s right to suitable instruction and support. If she does not succeed, she experiences a crisis. Through the exchange with the practice teacher and the tandem partner, she succeeds in revising misconceptions and, for example, in working on her understanding of her role.

Tina L: I can’t really remember the lesson, I only know that afterward I had such a bad conscience, somehow because I couldn’t get across what I actually wanted to teach the children, so that because of me they have no idea what they are supposed to do. […] and somehow I thought that the whole knowledge [is] somehow my responsibility, and I have to get that across in the 10 min. I would say that’s not my view anymore today. (TL_t2, 120–135)

In the 3rd year of study, a change can be observed in that TL represents her pedagogical convictions argumentatively to the outside world—also vis-à-vis evaluation authorities. She asserts to her practice coach that the examination of a 3rd grade student’s performance in a movement landscape is implemented in a way that avoids “pressure” or possible shaming. Until the end of her studies, her orientation toward recognition and appreciation, which she incorporated from her school days, guides her actions and documents her attempt to reduce the teacher’s power of determination. In addition, there are many other requirements (of cooperation, of differentiation, of assessment) that TL works on from the logic of the teacher’s perspective.

With the detachment from guidelines and foreign ideas of instructional design, a teacher habitus is already constituted at t3, which replaces the student habitus. As it turns out, this process takes place in accordance with stabilizing an own system of rules and designing and the solidifying pedagogical and didactic orientations.

Tina L: I think what I’m looking forward to when I look at the future now is finding my own system in the classroom and creating my own framework and setting the priorities for myself. And forcing the kids into it. @(.)@ (t3_TL, 382–385).

In comparison to her study entry and to the negative images of school and of the profession embedded in the pupil habitus, it can be summarized that for TL at the end of the study, the fundamental question of the feasibility and realizability of being able to follow her pedagogical orientations at a regular school seems to recede into the background or to have been clarified. Rather, it becomes apparent at t3 that the initial question of her fit with the school system has changed into an active (co‑)shaping of the system. The laughter (@@) in the last quotation indicates that the exercise of power she experienced as a pupil is reflected by the teacher she is today. The “joy” she gets from anticipating the new situation points to the self-assessment of sufficient agency to cope with the professional tasks.

### Case Noah Summer

Noah Summer (NS) is 24 years old at the beginning of study. After secondary school, he completes a commercial apprenticeship. Following the apprenticeship, he passed the vocational baccalaureate and the preliminary course of the University in order to gain admission to the study. His decision to become a teacher was made “on short notice” and through the attribution of a professional aptitude from the outside (“that I was good with children”). He completes his first internship in a 5th grade class. In the second half of his partner-school internship, he already substitutes in another school on two mornings. There, he completes his focus internship in his own class, where he shares the class leadership with a job partner. At the time of the last interview, it was clear that he would take up a 100% position as a classroom teacher after the summer.

It is evident from NS’s school narratives that he always considers his commitment and effort in relation to the output. During his primary school years, he learns that he succeeds in getting good grades without any effort (“I never had to do anything, my grade was always at least a five and a half”). Constitutive for him is an adapted behavior (“model pupil”), which is oriented to the anticipated expectations of the teacher.

Only later at the *Gymnasium* (sixth grade), his performance orientation is being irritated, but he lacks the ability to establish a connection between his own effort and the (lack of) grade-related success. In retrospection, NS formulates that he “didn’t check” the necessity of the effort, which, however, is not further specified. He counters the high-performance expectations at *Gymnasium* by transferring to the *Sekundarschule* and readjusting his expectations for yield.

Noah S.: My expectations of myself have always been very low, that is, a satisfactory grade and that’s it, that’s all I need. (NS_t3, 212–213).

His adaptations initially document a pupil habitus of “educational conformity” (Kramer 2014, p. 190), which becomes distant from the fulfillment of expectations when he changes school. When NS no longer succeeds in achieving the performance-related goal, he does not change his commitment, but modifies his self-expectation. This performance orientation also becomes action-guiding during his commercial apprenticeship, e.g., when he emphasizes that he “got through relatively easily.” A change first appears with the new emerging goal of university studies, for which he first has to pursue a qualification path.

Noah S.: Where I thought, come on, I want to study, I want to study and at some point the teaching profession crystallized out. That’s when my ambition started to take hold of me, and I really invested time and effort to achieve the goal and very often exceeded my expectations (.) so it was a kind of development in the sense that I realized that you have to do something if you want to achieve your goals. (NS_t3, 201–220).

On an explicit level, NS formulates a “change” in his efforts, a “checking” of necessities that seems to last in his studies. His increased commitment continues into his first year of study (“I gave my life there”). It seems that there is a high level of involvement with the concrete achievement of goals—at least at the action level. Thus, in his first internship, he announces to the practice teacher to “do as much as possible, on my own [to] get a realistic picture of the teaching profession.” In all three interviews with NS, the training-related orientation schema of wanting to experience the “reality” of the teaching profession is found. Against the background of his performance orientation in his pupil and apprenticeship years, it can be assumed that he is (also) concerned with estimating the required time effort and competencies. In his student habitus, however, what emerges most strongly is learning by doing, by gaining experience, which proves to be stable over the entire course of the study.

Noah S.: But I have the feeling that (.) you only learn anyway (.) when you really work as a teacher when you make these experiences. I have the feeling that experience is a little bit the be-all and end-all for a (.) teacher and (2) you can cram as much theory as you want now at a University for Applied Sciences or something (.) but (3) yes, finally the experience (1) must therefore, first of all, get the diploma” (NS_t2, 568–573).

With the relation of effort and commitment to the visible yield (“diploma”) during studies, a homology to the school-related learning and educational orientation as a primary-school pupil becomes apparent. NS’s frequently used concept of experience proves to be an additive stringing together and layering of lasting experiences, from which—according to his understanding of professionalization—teacher expertise is nurtured. In contrast, he considers the theoretical-reflexive part of the study to be irrelevant; the reflexive processing of the experiences in action is apparently secondary for him. The interplay between self-responsible activity, experiences of failure, and the lack of reflective processing of the experiences is documented in several internship situations that exceed his possibilities for coping and acting.

Noah S.: “It’s just a train that’s run into a wall at an unbelievably@ fast speed, and I’m standing there and, yes, praying that the lesson is finally over”.

The experience of his own limitations in the first internship, however, does not seem to cause NS to question his approach to teaching without the guidance of an experienced teacher. Rather, he looks for ways to close such crisis-like experiences without having to deal with them, e.g., by handing over a problematic child to his tandem partner or by leaving it to the practice teacher to solve the situations he has caused. In the subsequent partner school practicum, his orientation toward autonomous teaching action, carried out within a self-imposed framework, is lived out in self-selected projects. Here, too, he leaves annoying tasks to his tandem partner and justifies this with his time-consuming part-time job as a teacher at another school.

In the positive counter-horizon of his “internship in his own class” (PEK) as part of the focus internship in his final year of study, he reflects on the difference between supervised internship and his own employment as a teacher, again highlighting his performance orientation. While NS retrospectively experiences the first two practice phases as a test in which he “wanted to show what you can do” and at the end receives “a check mark” from the practice teacher, he perceives the focus internship in his own classroom as a time approaching the reality of the profession in which he no longer has the “university breathing down his neck.” Having to cope with all aspects of the teaching profession, he perceives as “valuable, simply because it does not cease in such a realistic setting, but it is part of it because you have to cope with it.” (t3_NS, 15–25) It seems that by leaving the probationary space and the institutionalized training framework of internship, one’s performance is measured for the first time in terms of self-assessment and emotional gain (“joy”).

Noah S.: And when you really work independently in a PEK then yes, just there, that was the moment when it really became clear to me, yes fully, I am now working alone, I am 100% involved and it works out I can do it and um yes, it gives me joy. (NS_t3, 107–111)

NS’s early teaching career (beginning in the third semester of study) nurtures the formation of a teacher habitus parallel to the student habitus. A central theme for him is the efficacy that a teacher’s role has on the classroom. He sketches the counter horizons of a relational “cool” teacher and a dictatorial “bully.” Both types of teachers are found in his narratives of his primary school biography as well as of his practice teachers.

His positively framed teacher habitus, as it emerges from the school narratives at the beginning of the study, is initially strongly oriented toward good relationship building. Based on his own experiences of limitation, the teacher habitus he strives for seems to transform. At the end of his studies, NS sketches a range of “being loose” and “military” styles in which a teacher must at times be able to move “as an actor.” Depending on the class, he puts on “two completely different faces”. This documents a technological understanding of professionalism that is also reflected in the additive layering of experience.

With reference to his student habitus, it can be stated that NS frames his studies from the beginning as practice situations that are realized primarily in the internships. He does not establish lines of connection between study content and practical experiences, nor does he establish concrete developmental goals that go beyond role-finding and classroom management. From the first internship on, the educational setting is framed as a training field rather than a developmental space where he could learn from professionally experienced teachers. For NS, feedback from instructors essentially represents a formal component; however, he does not attribute any significance to it for his professional development. Whether his skills are sufficient to cope with the job is measured for him exclusively by his success in the “real” world of work. He attests himself achievement of this goal at the end of his studies (“it works, I can do it”).

## Summary and discussion

As stated at the outset, the formation of teacher habitus is a “transformational process” (Kramer and Pallesen 2019, p. 80). The institution of school represents a field of social space for which “the acquisition of appropriate field-specific dispositions is required” (ibid., p. 79). It is therefore not surprising that students categorize the internship as that part of study in which they can build the necessary action-related knowledgebase for later professional practice. As Noah Summer’s case shows, he follows precisely this orientation of experiential layering. The university-based requirements and learning inventories are framed as a compulsory exercise. To “assimilate” all the requirements of a teacher—even if this leads to moments of crisis—allows him to feel the reality of the profession. Typical of this case is the fading out of certain demands by delegation to others, as well as the closing of crises. From an experiential theoretical perspective, the irritation of previous convictions and certainties is the developmental opportunity from which something new (Combe and Gebhard 2007) can emerge. However, through the closure and fading out of requirements, development potential remains unused. In the case of Summer, no change can be detected over the course of the study. It raises the question of what kind of student habitus is depicted here, considering Summers exclusive orientation toward learning in practice. At the end of his studies, Noah Summer seems to have reached the completion of becoming a teacher. His frame of reference is his experienced ability to cope with the (perceived) professional demands.

What stands out here is the lack of a reflexive distance, which is expressed in Summer’s subsumption logic and resistance to a scientific view of professional practice. According to Helsper (2018a, p. 128f.), this understanding of the profession, which is based on rules and on coping with demands of teaching, characterizes a “vocational teacher habitus”—in distinction to the “professional teacher habitus”.

As a representative of the latter, the case of Tina Lasnic is outlined. She emerges from her school biography and from the internship at the Montessori school—which she experiences as a positive counter horizon—with concrete pedagogical orientations. Subsequently, she tests the feasibility in the regular school system. In the course of her studies, a successively broadening perspective on all professional development tasks (Keller-Schneider and Hericks 2017) can be observed. The crises constituted by the diverse requirements are consistently dealt with, whereby the developmental orientation reconstructed at t2 becomes the guiding principle for action. Not least due to this habitual disposition, transformation steps from a reflexive student habitus to an already contouring teacher habitus are documented. The latter manifests itself in the detachment from specifications as well as in the anticipation of future establishment of her own teaching and working alliance concept, which is already recognizable in her student actions.

It further becomes clear from her case that the pedagogical orientations emerging from her pupil habitus are more than just the “raw forms and images of the teacher” (Kramer and Pallesen 2019, p. 81). Rather, it seems that despite their formulations of aspirations aligned to a variety of professionalism categories, the core theme deeply embedded in her pupil habitus remains central: *The recognition and appreciation of the pupils*’* individuality.*

The contrasts shown here can be supported and differentiated by the further 21 reconstructed cases. Thus, in a next step, it is possible to determine manifestations of student habitus in more detail, whereby these would have to be substantiated with explicit student requirements (e.g., written papers, examinations) as well as with concrete teaching-, interaction-, and subject-related aspects. It would also have to be further clarified how the transformation process from student to teacher habitus takes place. The depicted processes in the presented cases—normative quality assurance (Summer) and autonomy (Lasnic)—provide initial clues that should be further pursued.

In view of the diversity of the two cases presented, various questions arise for the teacher training. First, what is the concrete reason for the diversity and, consequently, how can it be addressed? If we look at the learning and achievement orientations of both students, the tendency of NS to reduce effort or to underachieve contrasts with TL’s high willingness to achieve, which was already apparent in her school years. At the same time, NS is found to be highly active and willing to take risks with a clear goal perspective, although this is limited to the action-practice study components and to the developmental tasks of role finding and class leadership. In comparison, TL works through demands in all four developmental tasks of role finding, facilitation, classroom management, and collaboration (Keller-Schneider and Hericks 2017). In her case, a high willingness to engage in crisis experiences was identified.

It can be concluded that understanding of learning based in one’s school biography leads to a homologous understanding of professionalization. As our own previous studies show, this orientation is very stable and not very irritable (Košinár 2014; Košinár and Laros 2020). The exclusion of professional-development tasks and certain requirements could be interpreted both strategically in terms of avoiding uncomfortable responsibilities or as a feature of overload (Košinár 2020), but perhaps also as a lack of knowledge.

For the university education the requirement arises, to find ways—by e.g., teaching theoretical models of professionalism—to irritate students existing occupation picture and to let possible hidden pictures emerge. In this way, students can be made aware of the necessity of working on all areas of professionalism. However, individual counseling, e.g., coaching or mentoring in the context of the internship courses, seems relevant for this purpose in order to work out individual development goals and to modify them during the course of study. In this context, opportunities for students to take responsibility for their own professionalization process should be promoted. Theories of professionalization and concepts can broaden the perspective and can also be used as a foil for analysis and reflection (Košinár 2018).

We see a greater challenge with regards to the initiation of a critical-reflection on the implicit orientations. Speaking from the theoretical perspective of teacher habitus, teachers’ actions are “essentially based on an incorporated habitus” (Helsper 2018b, p. 43) and only to a limited extent on the learning content and skills acquired in the course of studies and teacher training. It seems relevant to us as a next step to establish formats in teacher education within which school-biographical and familial imprints can be made reflexively accessible and to create space for “biographical-habitual self-reflection” (Helsper 2018a, p. 133), in which the willingness to question school- and teaching-related beliefs is encouraged (Košinár 2018). In this context, as the longitudinal analysis also shows, the study-entry phase is particularly suitable. In the transition process, access to the existing orientations seems most likely to be feasible.

## Footnotes

1 All Translations of quotations from German-language (literature or interviews) were made by the authors.

2 Here, a difference between a single-phase and a two-phases teacher education opens up, which is still to be discussed. The process described by Helsper (2018b) must (be able to) take place within the practical phases of the studies in the single-phase teacher education system.
